# Identification of suitable housing system for dairy cattle in North East Zone of Tamil Nadu, India, with respect to microclimate

**DOI:** 10.14202/vetworld.2017.1-5

**Published:** 2017-01-04

**Authors:** T. Sivakumar, P. T. Suraj, A. Yasotha, Jayashree Phukon

**Affiliations:** Department of Livestock Production Management, Madras Veterinary College, Tamil Nadu Veterinary and Animal Sciences University, Chennai, Tamil Nadu, India

**Keywords:** dairy cattle, micro-climate, roofing pattern

## Abstract

**Aim:**

To identify the suitable roofing pattern for dairy cattle in North East Zone of Tamil Nadu, India, based on micro climatic conditions.

**Materials and Methods:**

Initially, survey was conducted to identify and categorize the major housing patterns existing in the region for further detailed investigation. In total, 30 farmers/farms consisting of five housing types with six replicates were selected. Temperature and temperature humidity index (THI) were recorded using the maximum-minimum thermometer and digital thermo-hygrometers. The study was conducted for 1 year covering four seasons namely South West monsoon (June-August), North East monsoon (September-November), cold season (December-February), and summer season (April-May). The data were statistically analyzed using statistical package SPSS 17.

**Results:**

Animal shelters with cement sheets recorded the highest temperature (26.71±1.13°C) and THI (77.23±1.76) at 8.00 am, whereas the lowest temperature (24.83±1.17°C) and THI (74.54±1.72) were recorded in the thatched shed. There was significant difference (p<0.01) in temperature and THI at 8.00 am during South West monsoon and North East monsoon seasons between the housing types. During cold and summer seasons, there was no significant difference (p≥0.05) in the environmental variables among various shelter systems.

**Conclusion:**

Thatched housing is found to be the suitable one with respect to the climatic variables, followed by tile roof and metal roof. The cement sheet roofed housing is found to be the most unsuitable one in the region for dairy cattle.

## Introduction

Production and health of animals depend mostly on environment in which they live. A conducive environment for any farm livestock is the one that ensures not only optimal productivity but also meets the health and behavioral needs of the animals. The macro and micro environment have strong influence on farm animals with air temperature having the primary effect, and altered by wind, precipitation, humidity and radiation. Ideally, the impact of the thermal environment can be described in terms of effective ambient temperature which combines the various climatic factors. The extended periods of high ambient temperature coupled with high relative humidity compromise the ability of dairy animals to dissipate excess body heat in Indian climatic conditions [1,2].

In India, the upper temperature limit of comfort zone for optimum milk production is 27°C, about two degrees higher than the same reported in temperate countries [3]. This is perhaps because the crosses of exotic breed with native Indian breeds have adapted to climatic conditions in the country. However, the average annual temperature is higher than this upper limit in several parts of the country, particularly in South Eastern region comprising of the states of Andhra Pradesh and Tamil Nadu. Even though daily maximum temperatures exceeds 40°C for a few hours each day, cool nights help to lose the stored heat which allows the dairy cattle to produce at near optimal levels [4]. In a classical work, Johnson *et al*. and Zewdu *et al*. [5,6] reported that milk yield and dry matter intake exhibited significant declines when temperature humidity index (THI) reached 77. Animal productivity and efficiency are significantly influenced by environmental factors. Ambient temperatures above the thermal neutral zone are detrimental to lactation, growth, and reproduction in all livestock, but the effects on the dairy cattle are most economically severe [7-9]. Average daily milk yield/cow (kg) was reduced by 0.886 per unit increase of THI [10]. Studies conducted in New Zealand have demonstrated that the body temperature of dairy cows is related to air temperature and solar radiation [11].

The study was conducted to compare the different housing pattern used for dairy cattle and to find out the suitable one based on the climatic variables under which the productivity of dairy cattle is optimum in North Eastern zone of Tamil Nadu.

## Materials and Methods

### Ethical approval

Ethical approval was not necessary for this study as the study was based on the survey and no animals were involved in the study at any stage of the study.

### Location and geographical area under study

The study was conducted in Vellore and Villupuram districts of the North Eastern zone of Tamil Nadu. The North Eastern zone comprising the districts of Vellore and Villupuram are situated between 12° 55’ and 11° 57’ of North latitude and 79° 11’ and 79° 32’ East longitude with an average elevation of 77 km above mean sea level. The climate in this zone is basically semi-arid. The annual rainfall of the zone excluding hills varies from 800 to 1400 mm. The average maximum temperature ranges from 28.2 to 38.9°C and the minimum from 19.5 to 24.8°C. The study areas were selected based on the availability of highest cattle population, breedable cattle, and total milk production in these districts. Moreover, no other study of similar nature had been reported from these areas. The first phase of the research involved the data collection on the existing dairy cattle housing patterns in the study area. Initially, survey was conducted among 139 farmers with a structured questionnaire developed in consultation with the extension personnel and subject matter specialists. From the survey, the major housing patterns existing in the region were identified and categorized for further detailed investigation. Farmers with at least five cows were selected with the major types of housing pattern identified for conducting further field investigations.

### Type of housing and parameters recorded

In total, 30 farmers/farms (five housing types with six replicates) were selected from this agro-climatic zone based on the survey to identify the suitable housing system for dairy cattle. The five housing types were selected based on the type of roofing materials used. Those roofing materials were thatched, tile, metal, cement concrete, and open method of rearing. The maximum-minimum thermometer and digital thermo-hygrometers were installed in the dairy cattle sheds of various shelter types for measurement of the climatic variables. The climatic parameters such as maximum temperature (°C), minimum temperature (°C), temperature at 8.00 am (°C), temperature at 2.00 pm (°C), THI at 8.00 am, and THI at 2.00 pm were recorded.

### Duration of study

The duration of study was for 1 year and it was divided into four seasons as per [12] as South West monsoon (June-August), North East monsoon (September-November), Cold season (December-February), and Summer season (April-May). Data were collected separately from each farm for all the seasons.

### Statistical analysis

The collected data were statistically analyzed by one-way analysis of variances for finding out the differences between the groups using statistical package SPSS 17. The significance was tested using Duncan’s multiple range test [13].

## Results and Discussion

Season-wise climatic variables in various housing types in North Eastern zone are presented in Table-1. There was significant difference (p<0.01) during South West monsoon season between the housing types in temperature and THI at 8.00 am. Animal shelters with cement sheets recorded the highest temperature (26.71±1.13°C) and THI (77.23±1.76) at 8.00 am, whereas the lowest temperature (24.83±1.17°C) and THI (74.54±1.72) were recorded in the thatched shed.

**Table-1:** Mean±SD of season wise climatic variables in various housing types in North Eastern zone.

Season	Climatic variables	House type (n=15)					Over all	F value	p value

		Thatch	Tile	Metal	CC	Open			
South West monsoon	T_max_ (°C)	33.49±2.42	33.82±2.43	34.19±2.42	34.59±2.42	34.39±2.42	34.09±2.39	0.499	0.736
	T_min_ (°C)	24.66±1.17	24.86±1.17	24.96±1.17	25.26±1.17	24.37±1.17	24.82±1.18	1.225	0.308
	T at 8.00 am (°C)	24.83±1.17^ac^	26.04±1.17^b^	25.74±1.13^b^	26.71±1.13^c^	25.22±1.22^ab^	25.71±1.31	5.905	0.000
	THI at 8.00 am	74.54±1.72^abc^	76.30±1.72^b^	75.67±1.72^a^	77.23±1.76^c^	74.73±1.81^a^	75.69±1.98	6.153	0.000
	T at 2.00 pm (°C)	33.45±2.42	33.75±2.39	33.94±2.39	34.51±2.17	34.13±2.40	33.95±2.32	0.433	0.784
	THI at 2.00 pm	84.75±3.25	84.64±3.18	85.00±3.19	85.43±2.99	84.49±3.12	84.86±3.08	0.204	0.936
North East monsoon	T_max_ (°C)	31.91±2.46	32.24±2.46	32.61±2.46	33.01±2.46	32.81±2.46	32.51±2.43	0.483	0.748
	T_min_ (°C)	24.12±1.32	24.32±1.32	24.42±1.32	24.72±1.32	23.83±1.33	24.28±1.32	0.946	0.443
	T at 8.00 am (°C)	24.29±1.32^a^	25.46±1.31^bc^	25.13±1.32^abc^	26.09±1.32^c^	24.49±1.33^ab^	25.09±1.44	4.594	0.002
	THI at 8.00 am	74.68±2.03^a^	76.51±1.96^bc^	75.79±1.98^ab^	77.42±2.00^c^	74.60±1.98^a^	75.80±2.22	5.525	0.001
	T at 2.00 pm (°C)	31.87±2.46	32.18±2.46	32.37±2.46	33.05±2.46	32.56±2.46	32.40±2.43	0.481	0.749
	THI at 2.00 pm	84.45±3.47	84.52±3.40	84.87±3.42	85.54±3.37	84.45±3.33	84.77±3.33	0.286	0.886
Cold	T_max_ (°C)	30.32±1.93	30.67±2.00	30.77±2.01	31.35±2.07	31.15±2.13	30.85±2.01	0.598	0.665
	T_min_ (°C)	19.04±2.51	19.59±2.06	19.34±2.51	19.64±2.51	18.76±2.54	19.27±2.39	0.351	0.842
	T at 8.00 am (°C)	19.69±2.00	20.67±2.24	20.33±2.15	21.36±2.14	20.10±1.83	20.43±2.10	1.389	0.247
	THI at 8.00 am	67.00±3.54	68.59±3.93	67.92±3.76	69.67±3.77	67.48±3.17	68.13±3.67	1.225	0.308
	T at 2.00 pm (°C)	30.12±2.02	30.33±1.99	30.55±1.99	31.23±1.99	30.74±1.99	30.59±1.98	0.681	0.608
	THI at 2.00 pm	79.97±2.33	79.94±2.26	80.13±2.21	80.79±2.17	79.75±2.14	80.12±2.19	0.484	0.748
Summer	T_max_ (°C)	36.97±2.58	37.27±2.58	37.67±2.58	38.07±2.58	37.87±2.58	37.57±2.55	0.449	0.773
	T_min_ (°C)	25.09±2.63	25.33±2.62	25.39±2.63	25.69±2.63	24.79±2.63	25.26±2.57	0.248	0.910
	T at 8.00 am (°C)	25.26±2.63	26.46±2.62	26.14±2.58	26.97±2.50	25.40±2.62	26.05±2.60	1.154	0.339
	THI at 8.00 am	74.98±3.55	76.71±3.45	76.03±3.35	77.37±3.21	74.76±3.43	75.97±3.45	1.604	0.183
	T at 2.00 pm (°C)	36.66±2.28	36.60±1.89	37.02±2.09	37.91±2.36	37.62±2.58	37.16±2.25	1.004	0.411
	THI at 2.00 pm	87.28±2.72	86.21±2.16	86.79±2.44	87.67±2.59	86.63±2.87	86.92±2.55	0.734	0.572

THI: Temperature humidity index

There was significant difference (p<0.01) in temperature and THI at 8.00 am during South West monsoon and North East monsoon seasons between the housing types. However, during cold and summer seasons, there was no significant difference (p<0.05) in the above parameters. The THI was found to be below the critical mean of 72 [14,15] during morning 8.00 am in cold season in all the housing systems. During all other periods, it was above the recommended mean value indicating that the environmental stress in animals starts at morning hours of the day.

The minimum temperature recorded during all the four seasons in the zone was within the upper temperature limit of comfort zone for optimum milk production recommended by Dutt *et al*. [3], Fuquay [16], Mote [17]. However, the temperature at 2.00 pm was above this level in all the housing systems indicating the need for additional heat amelioration measures.

During North East monsoon season also, there was significant difference (p<0.01) between the housing types in temperature and THI at 8.00 am. Animal shelters with cement sheets recorded the highest temperature (26.09±1.32°C) and THI (77.42±2.00) at 8.00 am, whereas the lowest temperature (24.29±1.32°C) recorded in the thatched shed and lowest THI (74.60±1.98) recorded in the open.

In the North East monsoon season, the thatched housing system recorded lowest temperature at 8.00 am and 2.00 pm, and lowest THI at 8.00 am during South West monsoon, lowest T_max_, temperatures at 8.00 am and 2.00 pm during North East monsoon, lowest T_max_, temperatures at 8.00 am and 2.00 pm and lowest THI at 8.00 am during cold season, lowest T_max_ and temperature at 8.00 am. Hence, the thatched housing is found to be suitable one with respect to the climatic variables. Similarly, Bharambe *et al*. [18] concluded that thatched roof with paddy straw shed effectively ameliorates environmental temperature, humidity and THI during summer season in the Konkan region. Kamal *et al*. [19], Shekhawat and Chaudhary [20] reported that both thatched, mud plaster roof, and agro-net shade material were found to be the suitable roofing material for relieving the summer stress in a better way.

The tile roofed housings have shown the second lowest T_max_ during all the seasons of the year. This system has shown the least value for THI at 2.00 pm during summer season. Hence, tile roofed housing is also found to be suitable for the region with respect to the microclimate.

During cold and summer seasons, there was no significant difference (p≥0.05) in the environmental variables under various shelter systems under study in the North Eastern zone.

The metal roofed shelters were found to show the third position in T_max_ during all the four seasons. The THI at 2.00 pm was also on the higher side on the second position.

The cement sheet roofed housing has recorded the highest T_max_, T_min_ temperatures at 8.00 am and 2.00 pm, THI at 8.00 am and 2.00 pm in all the seasons. Hence, it is found to be the most unsuitable one in this region.

The open housed system has reported the lowest T_min_ during all the seasons due to increased heat dissipation during night; however at 2.00 pm it has recorded the second highest temperature after the cement sheet roofed housing. Hence, it cannot be recommended as a suitable one without the combination of shade during day time.

It is clear from the study that the morning and evening THI values exceeded critical value of 72 and ranged between 74.54±1.72 and 87.67±2.59 in all the seasons except cold season. The study also reveals that the dairy cattle in all the housing pattern were in mild stress during monsoon season also. Similar results were also reported by Kamal *et al*. [1] in cross bred calves. The present finding is also supported by Das [10], Khongdee [21] who found that the difference between maximum and minimum THI during the rainy season was lower indicating that the dairy cows were exposed to heat stress conditions more consistently during the rainy season.

In this study, the lowest minimum temperature (18.76±2.54) was observed in open type during cold season whereas highest maximum temperature (38.07±2.58) was recorded in cement concrete roof during summer. There was no significant difference between morning and evening temperature among different roofing types. Whereas, Jat *et al*. [22], Kamal *et al*. [23] recorded significantly (p<0.01) lower maximum temperature in shed with thatched and mud plaster roof than loose house with asbestos sheet and barn house. However, they observed the nonsignificant difference between morning and evening temperature with various roof structures similar to this study.

## Conclusion

The need arises to identify the region based suitable housing pattern for dairy cattle to diminish the challenges imposed by impending climate change. In this regard, the study was conducted to identify the suitable region wise housing system for dairy cattle. The result based on climatic variables in various housing system showed that the minimum temperature recorded during all the four seasons in the zone was within the upper temperature limit of comfort zone for optimum milk production. However, the temperature at 2.00 pm was above this level in all the housing systems indicating the need for additional heat amelioration measures. The result indicated that thatched roof, followed by tile and metal roof housing system are found to be suitable for the North East Zone of Tamil Nadu, India, with respect to the microclimate.

## Authors’ Contributions

TS has planned the research work for execution. PTS conducted the study in different housing systems. PTS and JP analyzed the data and performed statistical analysis. AY drafted and revised the manuscript under the guidance of TSK. All authors read and approved the final manuscript.
